# Relationship of serum estrogens and estrogen metabolites to postmenopausal breast cancer risk: a nested case-control study

**DOI:** 10.1186/bcr3416

**Published:** 2013-04-22

**Authors:** Roni T Falk, Louise A Brinton, Joanne F Dorgan, Barbara J Fuhrman, Timothy D Veenstra, Xia Xu, Gretchen L Gierach

**Affiliations:** 1 Hormonal and Reproductive Epidemiology Branch, Division of Cancer Epidemiology and Genetics, NCI, 6120 Executive Blvd, Bethesda, MD 20852, USA; 2 Fox Chase Cancer Center, 333 Cottman Ave, Philadelphia PA 19111, USA; 3 Department of Epidemiology Fay W Boozman College of Public Health, University of Arkansas for Medical Sciences, 4301 Markham St #820, Little Rock, AR 72205, USA; 4 Advanced Technology Program, Laboratory of Proteomics and Analytical Technologies, Frederick National Laboratory for Cancer Research, SAIC-Frederick, Inc., Frederick MD 21702, USA

## Abstract

**Introduction:**

Elevated levels of circulating estrogens are linked to breast cancer risk among postmenopausal women but little is known about the importance of estrogen metabolism. A recently developed liquid chromatography tandem mass spectrometry-based method (LC-MS/MS) measuring a panel of 15 estrogen metabolites (EM) has been evaluated in one study, linking high levels of 2-pathway metabolites relative to the parent estrogens to reduced breast cancer risk. We analyzed this panel of EM in a nested case-control study of postmenopausal breast cancer.

**Methods:**

Between 1977 and 1987, 6,915 women provided blood samples to the Columbia Missouri Serum Bank and were followed for incident breast cancer through December 2002. We studied 215 postmenopausal breast cancer cases and 215 matched controls who were postmenopausal and not using exogenous hormones at the time of blood draw. EM were examined individually, grouped by pathway (hydroxylation at the C-2, C-4 or C-16 positions of the steroid ring) and by ratios of the groupings. Logistic regression models controlling for matching and breast cancer risk factors were used to calculate quartile-specific odds ratios (ORs) and 95% CIs.

**Results:**

Significant elevated risks were not observed for individual EM, except for quartiles of 16-epiestriol (*P *trend = 0.07). The OR for total EM, the parent estrogens estrone and estradiol, and 2-pathway catechol EM (2-hydroxyestrone and 2-hydroxyestradiol) were elevated but the trends were not statistically significant. Among 2-pathway metabolites, risks for the highest levels of 2-hydroxyestrone-3-methyl ether and 2-methoxyestradiol were reduced; ORs for women in the highest versus lowest quartiles were 0.57 (95% CI = 0.33 to 0.99) and 0.53 (95% CI = 0.30 to 0.96), respectively. Overall, women with higher levels of 2-pathway EM had a reduced risk of breast cancer, which remained after accounting for levels of parent EM, 4-pathway EM and 16-pathway EM (all trends, *P *<0.11).

**Conclusions:**

Women with more extensive hydroxylation along the 2-pathway may have a reduced risk of postmenopausal breast cancer. Further studies are needed to clarify the risks for specific EM and complex patterns of estrogen metabolism. This will require aggregation of EM results from several studies.

## Introduction

**Figure 1:**
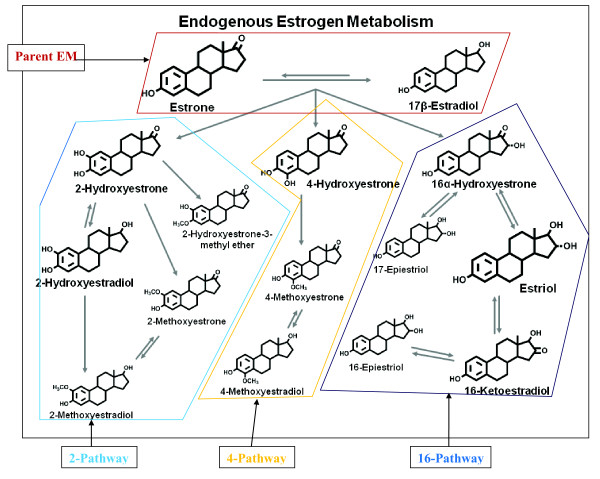
**Schematic of estrogen metabolic pathway**. Adapted from Fuhrman *et al*. [8].

Elevated levels of circulating estrogens are linked to postmenopausal breast cancer risk [1], but little is known about the role of specific estrogen metabolites (EM) or patterns of estrogen metabolism. Accruing evidence, primarily laboratory based, suggests substantial differences in the genotoxic, mutagenic, and proliferative activities of various EM and their contributions to mammary carcinogenesis [2-6]. Most breast cancer epidemiologic studies have evaluated immunoassays of only a few estrogens, specifically, estrone sulfate and the parent estrogens estrone and estradiol [1]. The development of a highly sensitive and reliable liquid chromatography tandem mass spectrometry method that simultaneously measures a panel of 15 EM in serum [7] has facilitated the exploration of the roles of individual EM and of pathways of estrogen metabolism that, until now, have not been satisfactorily measured in large population-based studies. This method measures the parent estrogens (estrone and estradiol, Figure 1), and their metabolites, derived through hydroxylation on either the A-ring or D-ring by cytochrome P450 enzyme isoforms, followed by methylation. On the A-ring, hydroxylation occurs predominately at the C2 position (2-pathway, Figure 1) and to a lesser extent, at the C4 position (4-pathway, Figure 1). Hydroxylation at the 16α position of the D-ring produces 16-pathway metabolites (Figure 1) [8].

Whether a panel of EM provides more discrimination of risk than conventional immunoassays of only the parent estrogens is not known. To date, the only study with prospectively collected bloods to have evaluated postmenopausal breast cancer risks associated with this panel of EM [8] found that women with high levels of 2-pathway metabolites had a reduced risk of breast cancer, as did those with more extensive methylation of the catechol metabolites (2- and 4-methoxyestrone, and 2- and 4-methoxyestradiol).

To corroborate these findings, we analyzed this panel of EM in sera drawn prospectively from participants in a nested case-control study of postmenopausal breast cancer identified from the Columbia Missouri Serum Bank Cohort. For a subset of the study participants, parent estrogens had been measured previously by an indirect radioimmunassay (RIA) [9], which also provided the opportunity to address the comparability of assay methods.

## Methods

### Study population

The Columbia Missouri Serum Bank was established as part of the National Cancer Institute's Biological Markers Project to identify serum markers of breast cancer, as has been described previously [9]. In brief, between 1977 and 1987, 6,915 women who were free of cancer other than non-melanoma skin cancer and living in or around Columbia, MO, USA, volunteered to provide blood samples, with most (90%) donating samples prior to 1980. Participants were identified through the Breast Cancer Detection Demonstration Project, the Women's Cancer Center Control Program at the University of Missouri hospital, and the Ellis Fischel Cancer Center. At the time of blood collection, information was obtained on known breast cancer risk factors, including age, weight, height, reproductive and menstrual history, family history of cancer, medical conditions and drug usage, including oral contraceptives and menopausal hormone therapy. Approximately 30% of the women donated multiple samples over the first 10 years of the study, with collections occurring on average one year apart. At each collection, 1.1 ml serum was aliquoted into glass vials and stored at -70°C. This study was approved by the National Cancer Institute (NCI) Institutional Review Board (IRB) and written informed consent was obtained from all participants.

Women were followed in two phases for incident breast cancer. The first follow-up, ending December 1989, identified 107 postmenopausal breast cancers, 70 of whom were also postmenopausal at blood collection. Breast cancer cases were identified by self report from a mailed questionnaire and medical records from the University of Missouri Hospital and Ellis Fischel Cancer Center, Missouri Cancer Registry and Missouri Department of Vital Statistics. A number of biomarker studies were conducted on the breast cancer cases and matched controls identified in this first follow-up including a study of serum estrogens and androgens [9] measured by RIA. In a second follow-up, 6,720 women (97% of the original cohort), who were thought to be alive and without a history of breast cancer or other cancer in the first follow-up phase, were contacted between 1999 and 2002, and an additional 197 breast cancer cases were identified. Thus, a total of 304 incident breast cancer cases were identified in this cohort, of whom 232 were postmenopausal at the time of blood donation and diagnosis. For this analysis, we excluded eight cases with limited blood remaining in the serum bank, seven who did not have a date of diagnosis, one diagnosed prior to blood draw and one lost to follow-up in the second phase of the study. The 215 breast cancer cases included in this study were individually matched to one of 215 postmenopausal controls who was alive and cancer-free at the age of the case diagnosis, with matching on type of menopause (natural, ovariectomy, hysterectomy at age 54 or later, and early hysterectomy (age <54)); age (+/- five years), season of blood draw, years since menopause (<1, 1 to <2, 2 to <3, 3 to <4, 4+) and time of day at blood collection (11 am to 1pm, 2 to 5 pm and 7 to 10 pm). Participants who reported using exogenous hormones at the time of blood donation were not eligible for this study. Additionally, 65 cases and 48 controls had RIA results for estrone, estradiol and estrone sulfate available from the first follow-up phase [9] and liquid chromatography tandem mass spectrometry (LC-MS/MS) assay measurements.

### Laboratory methods

Stable isotope dilution LC-MS/MS was used to quantitate15 EM including: estrone, estradiol, 2-pathway metabolites (2-hydroxyestrone, 2-methoxyestrone, 2-hydroxyestradiol, 2-methoxyestradiol and 2-hydroxyestrone-3-methyl ether); 4-pathway metabolites (4-hydroxyestrone, 4-methoxyestrone and 4-methoxyestradiol); and 16-pathway metabolites (16α-hydroxyestrone, estriol, 17-epiestriol, 16-ketoestradiol and 16-epiestriol). Details of the method have been published previously [7]. For this study, six labeled internal standards were used: deuterated 2-hydroxyestradiol, 2-methoxyestradiol and estriol (C/D/N Isotopes Inc, Pointe-Claire, QC, Canada); deuterated 16-epiestriol (Medical Isotopes Inc, Pelham, NH, USA); and ^13^C-labeled estrone and estradiol (Cambridge Isotope Laboratories, Andover, MA, USA).

In serum, this method detects 15 EM which circulate primarily as sulfated and/or glucuronidated conjugates. Beginning with the parent estrogens (estrone and estradiol, Figure 1), hydroxylation occurs on the A- or D-ring. On the A-ring, metabolism proceeds predominately at the C2 position (2-pathway, Figure 1), producing the 2-pathway catechols: 2-hydroxyestrone and 2-hydroxyestradiol. Methylation of these 2-catechol metabolites yields the 2-methoxycatechols: 2-methoxyestrone, 2-methoxyestradiol and 2-hydroxyestrone-3-methyl ether. To a lesser extent, metabolism on the A-ring proceeds at the C4 position (4-pathway, Figure 1) yielding the 4-catechol (4-hydroxyestrone) which may be further methylated to 4-methoxyestrone and 4-methoxyestradiol. Hydroxylation at the 16α position of the D-ring produces 16-pathway metabolites (Figure 1): 16α-hydroxyestrone, estriol, 17-epiestriol, 16-ketoestradiol and 16-epiestriol. All metabolites circulate, at least in part, as sulfated and/or glucuronidated conjugates to facilitate transport and excretion, while only five (estrone, estradiol, estriol, 2-methoxyestrone and 2-methoxyestradiol) also circulate in unconjugated forms. To capture the unconjugated forms, the serum sample was split into two aliquots, one to measure the total concentration of each of the 15 metabolites (that is, the sum of conjugated plus unconjugated forms); the other, to measure the unconjugated forms. To measure the sum of the conjugated plus unconjugated forms, an enzyme with sulfatase and glucuronidase activity is added to the samples to cleave any sulfate and glucoronide groups from the parent estrogens [7]. To measure the unconjugated forms only, addition of the enzyme is not included in the sample preparation steps. For those metabolites with both total and unconjugated measurements, the concentration of the conjugated form was calculated as the difference between the total and the unconjugated measurements. Sera from cases and matched controls were assayed in the same batch. Duplicate quality control (QC) samples from two women were placed in each batch, and samples in the batch were randomly ordered so that laboratory personnel were masked to study or QC status. Laboratory coefficients of variation were less than 5% for all individual estrogens and estrogen metabolites measured and less than 3% for estrone, estradiol, and estriol.

### Methodologic studies

In addition to exploring the etiologic role of EM in breast cancer, this cohort provided a unique opportunity to address a number of questions, including the temporal variability of this panel of EM in postmenopausal women and the comparability of these measurements to other assay methodologies. To study the first question, sera from 24 controls who provided bloods at two or more consecutive, annual screening visits were assayed, with bloods from each woman measured in the same batch. Of these, 22 had serum from three blood collections occurring one year apart; two had two blood collections, each one year apart. Additionally, we compared levels of circulating estrone, estradiol and estrone sulfate measured by RIA [9] to levels of total and unconjugated estrogen and estradiol obtained by LC-MS/MS in a subset of 68 cases and 45 controls.

### Statistical methods, case-control analysis

Initially, the distribution of each EM was analyzed to identify possible outliers and to apply appropriate data transformations. Non-parametric models were used to evaluate median differences in EM levels between cases and controls. Case-control differences between individual EM, pathway EM (for each of the 2-, 4- and 16-pathways, the sum of individual EM in the respective pathway, see Figure 1) and the ratios of these pathways were explored.

Unconditional logistic regression models were used to estimate quartile-specific odds ratios (ORs) and 95% CIs for individual EM, pathway EM and the ratios of the pathways. ORs controlling for matching factors and known breast cancer risk factors were calculated, with confounding addressed by including covariates that changed the effect estimates by more than 10% in the final model. The final model included: family history of breast cancer; body mass index (BMI); age at menarche; parity and age at first birth; age at blood collection; type of menopause; years between blood collection and menopause; season; and time of day of blood draw. For each EM, further adjustment for unconjugated estrone was made to assess whether levels of a given EM contributed to risk independent of parent levels; however, this did not alter OR estimates by >10% and results are not presented. The magnitude and patterns of risks from additional analyses, including using conditional logistic regression models, and excluding the 17 women whose breast cancer was diagnosed less than two years after blood donation, were comparable to the reported results and are not provided. No adjustment was made for multiple comparisons.

### Statistical methods, methodologic studies

We assessed EM consistency within a woman over two to three years using samples from controls with repeat blood collections, using nested analysis of variance (ANOVA) models to calculate the intraclass correlation coefficients (ICC). Pearson's correlation coefficients were used to assess comparability of RIA and LC-MS/MS measurements of the parent estrogens. All measurements were logarithmically transformed. For all analyses, all tests of significance were two-tailed and probability values of <0.05 were considered statistically significant. Analyses were performed using SYSTAT software [10] and forest plots were created with STATA [11].

## Results

### Study population

Table 1 presents the distribution of breast cancer risk factors and matching characteristics among cases and controls. Cases and controls were similar with respect to most known breast cancer risk factors including BMI, age at menarche, a family history of breast cancer, reproductive history and, among parous women, age at first pregnancy. However, parous cases had significantly fewer pregnancies (*P *<0.05) than parous controls. Most women in this study were naturally menopausal (71%), with the remaining having had an ovariectomy (13%) or hysterectomy (16%). The mean age at breast cancer diagnosis was 61.8 (range 50.8 to 76.8), with mean follow-up of 11.7 years (range 0.3 to 24.8 years). The age at blood collection for both groups was similar (mean of 59.4 and 59.8 for cases and controls, respectively).

**Table 1:** Distribution of cases and controls by breast cancer risk factors and matching characteristics; Columbia Missouri Serum Bank Cohort Breast Cancer Case-Control Study

			Cases		Controls		
				
			Number	%	Number	%	
				
			215	100	215	100	
**Breast cancer risk factors**							

Family history of breast cancer							
		yes	67	31.2	55	25.6	*P *= 0.199
							
	BMI						
		<25	89	41.4	95	44.2	
		25-29	87	40.5	80	37.2	
		30+	39	18.1	40	18.6	*P *= 0.778
		Mean (SD)	26.2 (+/- 3.9)		26.5 (+/- 4.8)		
							
	Ever smoke						
		yes	58	27.0	56	26.0	*P *= 0.270
							
	Age at menarche						
		<12	28	13.0	30	14.0	
		12	58	27.0	49	22.8	
		13	71	33.0	75	34.9	
		14+	58	27.0	61	28.4	*P *= 0.799
							

**Reproductive history**							

Never pregnant			36	16.7	34	15.8	*P *= 0.811
							
Number of prenancies^a^							
		1-2	102	57.0	83	45.9	
		3-4	57	31.8	66	36.5	
		5+	20	11.2	32	17.7	*P *= 0.002
							
Age at first pregnancy^a^							
		<20	24	13.4	34	18.8	
		20-24	98	54.7	85	47.0	
		25-29	38	21.2	41	22.7	
		30+	19	10.6	21	11.6	*P *= 0.415
		mean (SD)	23.9 (+/- 4.2)		24.2 (+/-4.7)		
							
^a^among parous women							
							
Age at menopause							
		<45	40	18.6	38	17.7	
		45 to 49	53	24.7	63	29.3	
		50 to 54	89	41.4	91	42.3	
		55+	20	9.3	10	4.7	
		unknown	13	6.0	13	6.0	*P *= 0.371
							
Years to breast cancer diagnosis (from blood draw)							
		<1	7	3.3			
		1 to <2	10	4.7			
		2 to <3	18	8.4			
		3 to <4	21	9.8			
		4-<5	9	4.1			
		5+	150	69.8			
							

**Matching factors**							

Age at blood collection							
		<55	60	27.9	62	28.8	
		55 to 59	58	27.0	49	22.8	
		60 to 64	45	20.9	43	20.0	
		65 to 69	33	15.3	35	16.3	
		70+	19	8.8	26	12.1	
							
Type of menopause							
		Natural	154	71.6	152	70.7	
		Ovariectomy	26	12.1	28	13.0	
		Hysterectomy after age 54	22	10.2	22	10.2	
		Early hysterectomy <age 54	13	6.0	13	6.0	
							
Years between menopause and blood collection							
		<1	6	2.8	6	2.8	
		1 to <2	15	7.0	17	7.9	
		2 to <3	10	4.7	8	3.7	
		3 to <4	9	4.2	9	4.2	
		4+	175	81.4	171	79.5	
		unknown			4	1.9	
							
Time of blood collection							
		11 am to 1 pm	82	38.1	80	37.2	
		2 pm to 5 pm	62	28.8	60	27.9	
		6 pm to 10 pm	71	33.0	71	33.0	
		unknown			4	1.9	

### EM distributions and correlations

Median and interdecile ranges (10% and 90% percentiles) of each EM are presented for cases and controls (Table 2). Among all study participants, levels of estriol, estrone and 2-hydroxyestrone were highest, accounting for approximately 73% of the total EM in circulation. Except for 2-methoxyestradiol, EM levels were not significantly different between cases and controls.

**Table 2:** Medians and inter-decile ranges, estrogen metabolites^a ^cases and controls, Columbia MO Serum Bank Cohort Breast Cancer Study

	Cases			Controls		
	
	Median	Inter-decile range		Median	Inter-decile range	
**Parent Estrogens**	**50%**	**10%**	**90%**	**50%**	**10%**	**90%**

Estrone						
total	252.76	108.7	676.4	226.76	96.5	842.7
conjugated	203.25	75.1	577.6	183.25	68.7	741.1
unconjugated	46.62	25.5	85.5	43.47	22.8	97.5
Estradiol						
total	26.80	11.4	72.0	22.94	10.1	105.7
conjugated	13.73	5.0	40.8	12.06	4.9	45.2
unconjugated	11.19	4.5	36.3	10.02	4.1	41.3
						

**2-Pathway**						

2-hydroxyestrone	75.30	64.4	136.5	73.92	64.7	155.0
2-hydroxyestradiol	12.21	12.2	46.3	22.96	12.5	50.3
2-methoxyestrone						
total	43.65	7.4	84.2	41.42	22.3	100.4
conjugated	23.85	7.4	55.0	25.15	9.2	59.4
unconjugated	16.38	6.7	43.5	15.33	5.7	42.5
2-methoxyestradiol						
total	13.53^	6.4	29.4	15.68	6.9	37.6
conjugated	9.91^	3.7	25.7	11.40	4.3	36.0
unconjugated	2.51	1.0	7.9	2.16	0.9	8.0
2-methoxyestrone,3-methyl ether	5.63	1.9	16.9	6.58	2.2	16.5
						

**4-Pathway**						

4-hydroxyestrone	19.99	14.1	33.2	17.61	13.4	36.7
4-methoxyestrone	3.48	1.0	7.4	3.60	1.3	7.3
4-methoxyestradiol	1.89	1.0	5.3	1.97	1.0	5.5
						

**16-Pathway**						

16α-hydroxyestrone	37.87	27.8	72.9	37.25	27.5	85.0
Estriol						
total	283.29	167.2	580.9	271.98	163.4	624.7
conjugated	245.97	131.8	536.9	227.52	126.4	598.7
unconjugated	32.69	8.1	84.8	26.71	7.8	73.7
16-ketoestradiol	32.05	20.6	64.0	31.59	19.3	76.7
16-epiestriol	5.97	2.3	20.5	5.50	1.9	19.0
17-epiestriol	2.21	1.1	8.1	2.33	1.0	7.5
						
**Total EM**	837.91	540.4	1777.9	802.76	512.7	2151.8

^a^pmol/l. ^*P *<0.05, Wilcoxon rank sum test of differences between cases and controls.

Among controls, levels of the parent estrogens, estrone and estradiol, were highly correlated with each other (rho = 0.75, results not presented); with estrone catechols (2-hydroxyestrone and 4-hydroxyestrone; rho ranged from 0.62 to 0.73) and with some 16-pathway EM (16α-hydroxyestrone, estriol, and 16keto estradiol; rho ranged from 0.65 to 0.90). Correlations between the parent estrogens and the methoxycatechols were lower (rho ranged from 0.22 to 0.43), while correlations between the methoxycatechols themselves, or between the methoxycatechols and either the 16-pathway EM or the 2- and 4-catechols were low to moderate (rho ranged from 0.09 to 0.43).

### Odds ratio results

#### Individual estrogen metabolites

**Figure 2:**
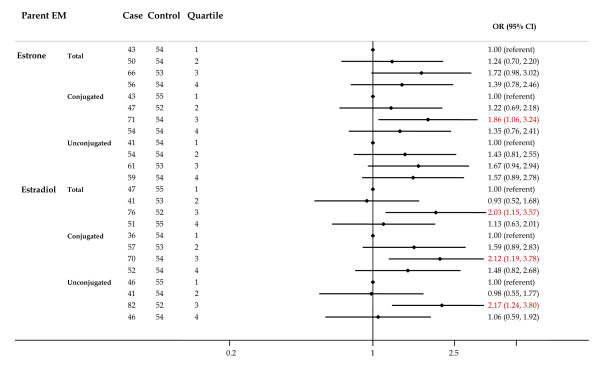
**Forest plot of OR and 95% CI for parent estrogens**. The center mark on each line corresponds to the odds ratio (OR) estimate and the length of the line corresponds to the confidence interval. Quartile cutpoints are based on the control distribution. For the referent quartile, only a mark at 1.0 is presented. ORs are adjusted for family history of breast cancer; BMI; age at menarche; parity and age at first birth; age at blood collection; type of menopause; years between blood collection and menopause; and time of day of blood draw. BMI, body mass index.

For the parent estrogens, risks for total, conjugated and unconjugated estrone and conjugated estradiol were elevated in all quartiles relative to the lowest quartile, but no trends were observed (Figure 2), and only the ORs for the third quartiles of estrone and of estradiol were statistically significant.

**Figure 3:**
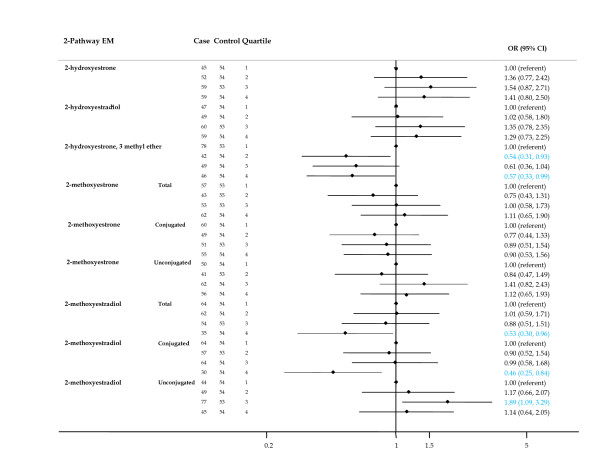
**Forest plot of OR and 95% CI for 2-pathway estrogen metabolites**. The center mark on each line corresponds to the odds ratio (OR) estimate and the length of the line corresponds to the confidence interval. Quartile cutpoints are based on the control distribution. For the referent quartile, only a mark at 1.0 is presented. ORs are adjusted for family history of breast cancer; BMI; age at menarche; parity and age at first birth; age at blood collection; type of menopause; years between blood collection and menopause; and time of day of blood draw. BMI, body mass index.

Among the 2-pathway metabolites (Figure 3), risks for the catechols (2-hydroxyestrone and 2-hydroxyestradiol) were non-significantly elevated, while the ORs for some methoxycatechols were reduced. Higher concentrations of 2-hydroxyestrone, 3-methyl ether and total 2-methoxyestradiol were associated with significantly reduced breast cancer risk (*P *trend for both <0.05). ORs for the highest versus lowest quartiles were 0.57 (95% CI = 0.33 to 0.99) and 0.46 (95% CI = 0.25 to 0.84) for 2-hydroxyestrone, 3-methyl ether and total 2-methoxyestradiol, respectively.

**Figure 4:**
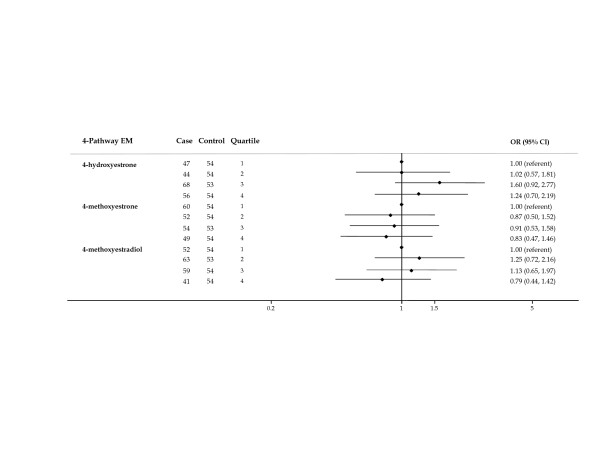
**Forest plot of OR and 95% CI for 4-pathway estrogen metabolites**. The center mark on each line corresponds to the odds ratio (OR) estimate and the length of the line corresponds to the confidence interval. Quartile cutpoints are based on the control distribution. For the referent quartile, only a mark at 1.0 is presented. ORs are adjusted for family history of breast cancer; BMI; age at menarche; parity and age at first birth; age at blood collection; type of menopause; years between blood collection and menopause; and time of day of blood draw. BMI, body mass index.

Of the 4-pathway metabolites (Figure 4), risks for moderate and high levels of the catechol 4-hydroxyestrone were not significantly elevated and no trend was apparent. Declining risk for higher levels of 4-methoxyestrone was suggested (*P *trend = 0.099).

**Figure 5:**
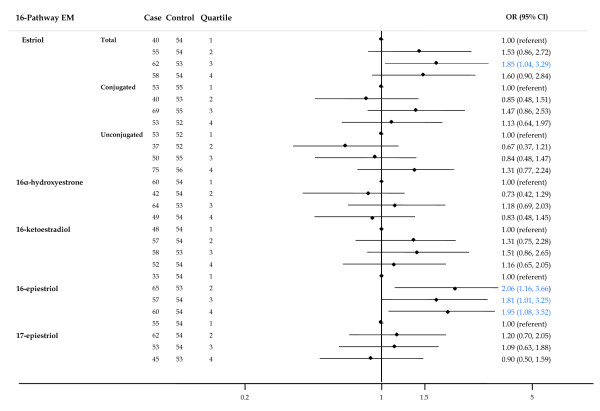
**Forest plot of OR and 95% CI for 16-pathway estrogen metabolites**. The center mark on each line corresponds to the odds ratio (OR) estimate and the length of the line corresponds to the confidence interval. Quartile cutpoints are based on the control distribution. For the referent quartile, only a mark at 1.0 is presented. ORs are adjusted for family history of breast cancer; BMI; age at menarche; parity and age at first birth; age at blood collection; type of menopause; years between blood collection and menopause; and time of day of blood draw. BMI, body mass index.

In the 16-pathway (Figure 5), borderline significant trends (*P *= 0.099) of increasing risk with increasing concentration were observed for total estriol and 16-epiestriol.

#### Pathways

**Figure 6:**
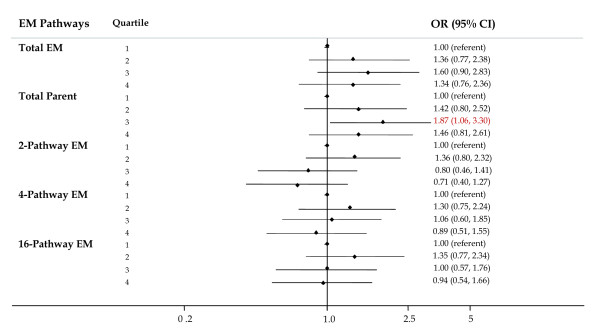
**Forest plot of OR and 95% CI for parent, 2-, 4-, and 16-pathway estrogen metabolite groups**. The center mark on each line corresponds to the odds ratio (OR) estimate and the length of the line corresponds to the confidence interval. Quartile cutpoints are based on the control distribution. For the referent quartile, only a mark at 1.0 is presented. ORs are adjusted for family history of breast cancer; BMI; age at menarche; parity and age at first birth; age at blood collection; type of menopause; years between blood collection and menopause; and time of day of blood draw. BMI, body mass index.

For this component of the analysis, EM in a given pathway was summed. For total EM (sum of all EM) and the parent estrogens (estrone plus estradiol), ORs were elevated but not significant at all levels and no trends were observed (Figure 6). Declining risks with increasing levels of 2-pathway EM were suggested (*P *trend = 0.099), but the individual risks were not significant. No associations were observed for the 4-pathway or 16-pathway EM.

#### Ratios

**Figure 7:**
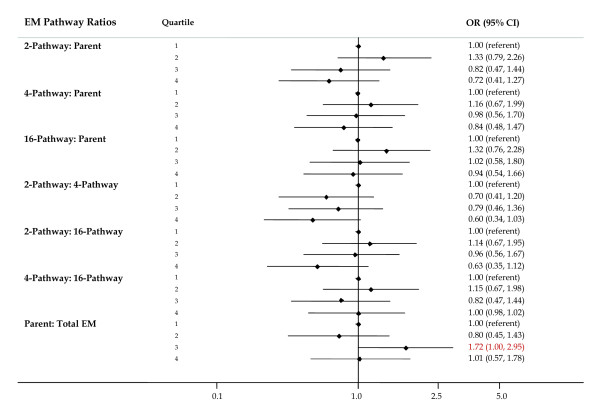
**Forest plot of OR and 95% CI for ratios of pathway estrogen metabolites to parent estrogen metabolites and other pathway estrogen metabolites**. The center mark on each line corresponds to the odds ratio (OR) estimate and the length of the line corresponds to the confidence interval. Quartile cutpoints are based on the control distribution. For the referent quartile, only a mark at 1.0 is presented. ORs are adjusted for family history of breast cancer; BMI; age at menarche; parity and age at first birth; age at blood collection; type of menopause; years between blood collection and menopause; and time of day of blood draw. BMI, body mass index.

Ratios of pathways were studied to explore the relative contribution of one pathway to breast cancer risk while accounting for either the precursor estrogens (parent estrogens) or another pathway. Relative to levels of the parent EM, women with higher levels of 2-pathway EM were at lower risk (OR for quartile 4 versus quartile 1 = 0.72 (95% CI = 0.41 to 1.27)), but the trend was not significant (*P *trend = 0.11, Figure 7). Risks for elevated levels of 4-pathway EM relative to parent EM showed a similar pattern of non-significantly reduced risks, but the trend was not significant. No consistent patterns of risk were observed for the ratios of 16-pathway to parent EM.

ORs for women with high versus low levels of the ratio of 2-pathway to 4-pathway EM and 2-pathway to 16-pathway EM were non-significantly reduced and trends were suggested (*P *= 0.10, Figure 7; OR highest quartile = 0.60 (95% CI = 0.34 to 1.03) and 0.63 (95% CI = 0.35 to 1.12) for the ratio of 2-pathway to 4-pathway and to 16-pathway EM, respectively).

ORs for the catechols (sum of 2-hydroxyestrone, 2-hydroxyestradiol and 4-hydroxyestrone) and methoxycatechols (sum of 2-methoxyestrone, 2-methoxyestradiol, 4-methoxyestrone, 4-methoxyestradiol and 2-hydroxyestrone, 3-methyl ether) were for the most part not statistically significant, and no trends were observed (data not shown). No patterns were observed for ratios of the catechol EM to methoxycatechol EM.

### Temporal variability of estrogen metabolites

Overall, among the 24 non-case women with two or more consecutive annual blood draws, the mean percent changes from one year to the next for estrone and estradiol were modest (2.1% and 8.8%, respectively). However, temporal differences were observed for the other EM. The ICCs for estrone and estradiol were relatively high (72% and 65%, respectively; Additional file 1), while for the 16-pathway EM, ICCs were moderate (35% to 53%). For the remaining EM in the 2- and 4-pathways, ICCs were low, ranging from 10% for 2-methoxyestrone to 38% for 2-hydroxyestrone.

### Comparison of estrogen levels by RIA and LC-MS/MS

**Figure 8:**
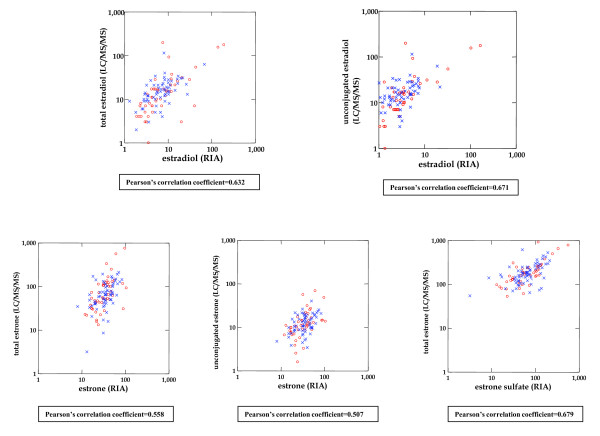
**Comparison of estrone and estradiol measurements by LC-MS/MS and RIA**. The top graphs compare levels of total and unconjugated estradiol measured by LC-MS/MS (y axis) to estradiol measured by RIA (x axis). The two leftmost graphs on the bottom compare levels of total and unconjugated estrone (by LC-MS/MS), respectively, to estrone (by RIA); the rightmost bottom graph compares total estrone (LC-MS/MS) to estrone sulfate (RIA). All axes are logarithmically scaled. LC-MS/MS, liquid chromatography tandem mass spectrometry; RIA, radioimmunoassay.

Figure 8 compares levels of total and unconjugated estradiol as measured by LC-MS/MS (y axis, top left and right plots, respectively) against RIA measures of estradiol (x axis). Pearson's correlation coefficients were moderately high, being 0.63 and 0.67, respectively. The bottom plots compare assay methods for estrone, with the two left plots comparing LC-MS/MS levels of total and unconjugated estrone, respectively (y axis) to RIA measured estrone (x axis) and the right-most plot of total estrone (LC-MS/MS, y axis) and estrone sulfate (RIA, × axis). Correlations for total and unconjugated estrone with RIA measured estrone were slightly weaker (Pearson's rho = 0.56 and 0.51, respectively) but levels of LC-MS/MS estrone compared favorably with RIA measured estrone sulfate (rho = 0.68).

## Discussion

In this nested case-control study of postmenopausal breast cancer and patterns of estrogen metabolism, increased risks for individual EM were not observed except for 16-epiestriol. Risks for estrone, estradiol and the 2-pathway catechols, 2-hydroxyestrone and 2-hydroxyestradiol, were non-significantly elevated at all levels of exposure, while risk reductions were suggested for higher levels of some 2-pathway methoxyestrogens, including 2-hydroxestrone 3-methyl ether, and conjugated and total 2-methoxyestradiol. Analysis of the pathways provided information beyond that contributed by parent estrogens alone since women with higher levels of 2-pathway EM overall tended to have a reduction in breast cancer risk, which persisted even after accounting for the contribution of their precursor hormones, estrone and estradiol. Similarly, relative to levels of both 4-pathway and 16-pathway EM, women with higher levels of 2-pathway metabolites were at lower, albeit not statistically significant, risk than those with low 2-pathway EM. Taken together, these findings suggest that women with more extensive estrogen metabolism along the 2-hydroxylation pathway may have a reduced risk of postmenopausal breast cancer.

To date, the only study of postmenopausal breast cancer to report findings from this panel of estrogens observed a similar protective effect among women with high 2-pathway metabolism [8]. In that study, unlike ours, high levels of both unconjugated estradiol and unconjugated estrone were significantly linked to elevated breast cancer risk. While we found elevated risks for high levels of unconjugated estrone, no trends were apparent and no association was observed for unconjugated estradiol. Additionally, the ranges of EM values among our postmenopausal women were much broader than those observed in the earlier study. Reasons for these differences are not clear; however, the state of current efforts to study the relationship between breast cancer risk and various EM is reminiscent of the early 1990s, when cohort studies began investigating levels of circulating sex steroid hormones. At that time, studies were relatively small (the number of cases by cohort ranged from 29 to 310), and most, but not all, linked elevated estrogens to breast cancer risk. It was not until results were pooled [1] that elevated levels of estrone and estradiol were conclusively linked to a doubling of breast cancer risk. We note that the ranges and absolute levels of estrone and estradiol in our study are comparable to levels observed in the studies that contributed to the pooled analysis, where conventional RIA assays were used [1,12].

Our study had a number of potential limitations. Unlike most studies of postmenopausal breast cancer, our cases were not more likely than controls to be overweight or obese, potentially limiting the generalizability of our findings. In addition, hormone receptor status could only be obtained on a fraction of the cases in this study. Serum samples were stored in glass vials at -70°C for more than 30 years and the consequence of such long-term storage on sex hormones is not known. Sample evaporation and degradation do not appear to have influenced measured EM levels in this study, since as discussed, the ranges of individual EM values were quite broad, and for those estrogens commonly measured by commercial laboratories, absolute levels were consistent with normative values [13,14]. Ironically, initiating this study in the late 1970s/early 1980s may have been an advantage, since menopausal hormone therapy was not highly recommended for postmenopausal women at that time. Although we excluded women currently using exogenous hormones, our EM values are not likely to have been contaminated by recent hormone use. The relatively small size of this study did limit our power to detect significant risks for individual EM and to disentangle the effects of complex patterns of estrogen metabolism. Thus, while it is not surprising that few of the individual risks or the trends reached statistical significance, our finding that women with more extensive 2-pathway metabolism may be at reduced risk of postmenopausal breast cancer does replicate results from the one previous postmenopausal breast cancer study of this EM panel [8].

While the primary intent of this study was to evaluate whether this panel of EM is informative with regard to breast cancer risk, it is the first study to explore features of this panel that are pertinent to epidemiologic research. For instance, in order to reflect adequately a woman's long-term adult hormone profile, EM measurements must be relatively stable from one year to the next. The year-to-year reproducibility of parent estrogens in postmenopausal women using RIAs has been conflicting [15-19], with ICCs ranging from 36% to 68% for estradiol and from 57% to 74% for estrone. In our study, multiple blood collections in the first years after enrollment allowed us to assess the temporal variability of this panel of EM among controls who contributed two or more bloods at yearly intervals. Our finding of relatively high ICCs for estradiol and estrone (≥65%) support the use of a single measurement by LC-MS/MS to categorize long term levels of these estrogens in postmenopausal women. However, for the other EM, where ICCs ranged from 10% to 50%, a single measure may not characterize long-term levels very well and may have contributed to our lack of findings for these EM by attenuating the observed risks. Additionally, in a subset of study participants for whom estrone and estradiol were measured using both RIA and LC-MS/MS methods, the levels correlated favorably. Compared to levels of estradiol measured by RIA where extraction via celite chromatography was applied before the assay was used [9], levels of total and unconjugated estradiol (LC-MS/MS) were well correlated. For estrone, the correlations were slightly lower, but total estrone measured by LC-MS/MS (which is the sum of unconjugated, sulfated and glucuronidated estrone) correlated highly with RIA measures of estrone sulfate.

Elevated levels of circulating estrogens have been convincingly linked to breast cancer risk among postmenopausal women [1,18], yet the molecular mechanisms underlying this link are not entirely clear, and even less is known about the importance of patterns of estrogen metabolism or of specific EM in postmenopausal breast carcinogenesis. Proposed mechanisms underlying this association include observations that some metabolites enhance the proliferation of breast epithelial cells mediated by estrogen receptor activity, and/or act as substrates for conversion into DNA- damaging compounds [2-4,20,21]. It is well recognized that levels of circulating estrogens in postmenopausal women, particularly estradiol, may be influenced by host characteristics such as BMI, lifestyle and dietary factors [12,21]; however, it is not known how or whether these factors alter estrogen metabolism. The development of laboratory methods to measure potential DNA-damaging metabolites in sera from large epidemiologic studies is a step forward in understanding the mechanisms of estrogen metabolism in breast carcinogenesis. This may help identify women who may benefit from strategies that alter estrogen metabolism through lifestyle and/or chemopreventive endocrine therapies. For now, it is not clear whether measuring specific EM provides more insight into breast cancer etiology than studies relying only on measures of the parent estrogens alone, although our findings, along with those from one prior study, suggest that women with more extensive estrogen metabolism, particularly along the 2-pathway, may have a reduced risk of postmenopausal breast cancer.

## Conclusions

Further investigations are needed to corroborate whether women with more extensive estrogen 2-pathway estrogen metabolism are at reduced risk of postmenopausal breast cancer and to identify whether breast cancer risk is associated with specific EM or other complex patterns of estrogen metabolism. This will require the aggregation of EM results from several studies. Additionally, efforts to replicate the temporal variability of the metabolites are needed to evaluate whether measures from a single blood collection are sufficient for meaningful risk assessment.

## Abbreviations

ANOVA, analysis of variance; BMI, body mass index; EM, estrogen metabolites; ICC, intraclass correlation coefficient; IRB, Institutional Review Board; LC-MS/MS, liquid chromatography-tandem mass spectrometry; NCI, National Cancer Institute; OR, odds ratio; QC, quality control; RIA: radioimmunoassay.

## Competing interests

The authors declare that they have no competing interests.

## Authors' contributions

All the authors made substantial contributions to the study design, conduct of the hormone assays or the analysis and interpretation of data. RTF and GLG conceived and designed the study, coordinated the research, provided guidance throughout the study process, performed the analysis, participated in interpretation of the data and drafted the manuscript. XX and TDV carried out hormone assays. JFD participated in the study design and sample acquisition, participated in the interpretation of the data and helped draft the manuscript. LAB and BJF participated in the analysis and interpretation of the data and helped draft the manuscript. All authors were involved in revising the manuscript, and have read and approved the final manuscript.

## Supplementary Material

Additional file 1 **Intraclass Correlation Coefficients of EM. Description: Temporal variability in EM measurements from 24 women who donated bloods annually over a three year period**. Click here for file
