# Supplementary material for: Relationship of serum estrogens and estrogen metabolites to postmenopausal breast cancer risk: a nested case-control study
Source: Breast Cancer Res. 2013 Apr 22;15(2):R34. doi: 10.1186/bcr3416 (PMC4053199; doi:10.1186/bcr3416)
Supplement: Additional file 1 — Intraclass Correlation Coefficients of EM. Description: Temporal variability in EM measurements from 24 women who donated bloods annually over a three year period. [file bcr3416-S1.DOC]

**Additional File 1:** **Intraclass Correlation Coefficients of EM**

|  | |
| --- | --- |
|  |  |

|  |  |  | **ICC (%)** |  |
| --- | --- | --- | --- | --- |
|  |  | | **N=24** |  |
|  |  | |  |  |
|  | **Parent Estrogens** | |  |  |
|  |  | **Estrone** | 71.7 |  |
|  |  | **Estradiol** | 64.8 |  |
|  | **16-Pathway** | |  |  |
|  |  | **16α-hydroxyestrone** | 43.1 |  |
|  |  | **Estriol** | 48.3 |  |
|  |  | **16-ketoestradiol** |  |  |
|  |  | **16-epiestriol** | 53.4 |  |
|  |  | **17-epiestriol** | 35.1 |  |
|  | **2-athway** |  |  |  |
|  |  | **2-hydroxyestrone** | 37.7 |  |
|  |  | **2-hydroxyestradial** | 27.4 |  |
|  |  | **2-hydroxyestrone, 3 methyl ether** | 14.1 |  |
|  |  | **2-methoxyestrone** | 9.6 |  |
|  |  | **2-methoxyestradiol** | 33.1 |  |
|  | **4-Pathway** |  |  |  |
|  |  | **4-hydroxyestrone** | 32.4 |  |
|  |  | **4-methoxyestrone** | 23.3 |  |
|  |  | **4-methoxyestradiol** | 33.3 |  |
|  | **Unconjugated EM** | |  |  |
|  |  | **Estrone** | 22.3 |  |
|  |  | **Estradiol** | 64.9 |  |
|  |  | **2-methoxyestrone** | 0.01 |  |
|  |  | **2-methoxyestradiol** | 52.2 |  |
|  |  | **Estriol** | 42.2 |  |

**2-Pathway**

**4-Pathway**
